# Dyke-Davidoff-Masson syndrome

**DOI:** 10.11604/pamj.2021.39.256.30993

**Published:** 2021-08-20

**Authors:** Moli Jai Jain, Rakesh Krishna Kovela

**Affiliations:** 1 Department of Cardiovascular and Respiratory Physiotherapy Sciences, Ravi Nair Physiotherapy College, Datta Meghe Institute of Medical Sciences, Wardha, Maharashtra, India,; 2 Department of Neuro-physiotherapy, Ravi Nair Physiotherapy College, Datta Meghe Institute of Medical Sciences, Wardha, Maharashtra, India

**Keywords:** Dyke-Davidoff-Masson syndrome, mirror movement, hand rehabilitation

## Image in medicine

We are presenting a case of 15-year-old male patient presented with weakness of right upper and lower limbs, difficulty lifting objects with the right hand. He was born full term to non- consanguineous parents with no significant antenatal or perinatal history. On examination there was hemi-atrophy of the right side of the body with simultaneous involuntary movements of contralateral hand accompanying voluntary movement of the ipsilateral side. Neurological examination reveals right side spastic hemiparesis with upper limb more affected than lower limb. Tendon reflexes were brisk on affected side with extensor plantar response. Mini-mental status examination score was 26/30 suggestive of mild cognitive impairment. Other systemic examinations were within normal limits. Plain and contrast magnetic resonance imaging (MRI) of brain findings reveal left cerebral hemi-atrophy with thinning of the ipsilateral grey matter cortex, reduced volume of the underlying white matter, enlargement of the left lateral ventricle and reduced size of ipsilateral left cerebral peduncle is noted. Along with slight compensatory thickening of the ipsilateral skull vault is seen. Findings confirm diagnosis of Dyke-Davidoff-Masson Syndrome. He was under medical management along with physiotherapy rehabilitation focussing majorly on hand rehabilitation.

**Figure 1:**
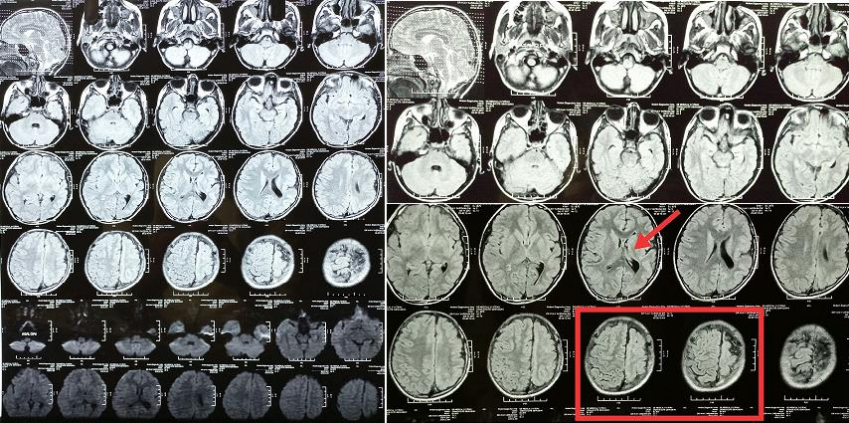
brain MRI findings shows left cerebral hemi-atrophy with thinning of the ipsilateral grey matter cortex, reduced volume of the underlying white matter (red box), enlargement of the left lateral ventricle (red arrow)

